# Significance of *HIF-1α* Expression and *LOXL-2* Localization in Progression of Oral Squamous Cell Carcinoma

**DOI:** 10.31557/APJCP.2021.22.2.341

**Published:** 2021-02

**Authors:** Anshul Bharti, Aadithya B Urs, Priya Kumar

**Affiliations:** *Department of Oral Pathology, Maulana Azad Institute of Dental Sciences, India.*

**Keywords:** Oral, squamous cell carcinoma, HIF-1α, LOXL-2

## Abstract

**Backgroud:**

In the microenvironment of Oral Squamous Cell Carcinoma (OSCC), Hypoxia-inducible transcription factor 1 (HIF-1) is a very important chemical mediator in the microenvironment of OSCC through which cells respond to hypoxia. LOXL-2 participates in ECM remodelling, and also in regulating epithelial-to-mesenchymal transition, epithelial cell polarity and differentiation.

**Aim/material and methods:**

The present study was conducted on 90 histopathologically proven cases of OSCC to ascertain the role of HIF-1α and LOXL-2 in OSCC. Immunoexpression of both HIF-1α and LOXL-2 was analyzed both quantitatively and qualitatively and compared with tumor stage, nodal stage, clinical stage, and histological grade.

**Results:**

Tumor stages and nodal stages had significant correlation with HIF-1α expression and localization of LOXL-2 immunoexpression respectively.

**Conclusion:**

This is probably the first study to analyze LOXL-2 localization in OSCC. Alteration in the immunoexpression of LOXL-2 from nuclear to cytoplasmic and HIF-1α immunoexpression might be an important factor in progression of OSCC.

## Introduction

Oral Squamous Cell Carcinoma (OSCC) represents 30% of head and neck carcinoma and is the fifth most common carcinoma in males and the eighth most common carcinoma in females worldwide (Harris, 2002). The OSCC microenvironment consists of tumor cells, different stromal cells, and the extracellular matrix (ECM) (Emon et al., 2018). Hypoxia in the tumor microenvironment results because of high energy and oxygen consumption. Hypoxia-inducible transcription factor 1 (HIF-1) is a very important chemical mediator through which cells respond to hypoxia in the tumor microenvironment (Harris, 2002). HIF-1α leads to increased expression of angiogenic markers, which are important for successful growth, invasion, and metastasis of a tumor (Emon et al., 2018). Oral cancer cells respond to hypoxia very strongly, and thus lead to increased VEGF expression and protein synthesis as an autocrine growth factor leading to increased angiogenesis and tumor growth (Shang et al., 2006).

Tumor microenvironment also comprises of cancer associated fibroblasts (CAFs). CAFs upregulate lysyl oxidase and thus, the stiffness of the stromal tissue increases (Emon et al., 2018). LOX protein family has five members: LOX and Lox-like (LOXL), LOXL1, 2, 3, and 4 (Canesin et al., 2015). Disturbance in ECM remodeling occurs in pathological conditions such as cancer and fibrosis. LOXL-2 has an important role in activation of fibroblasts in the tumor microenvironment. LOXL-2 participates in ECM remodeling, and also in regulating epithelial-to-mesenchymal transition, epithelial cell polarity, and differentiation. (Barker et al., 2013) Some studies also show that pro-metastatic function of LOXL-2 is independent of its role in ECM remodeling; others show that increased LOX secretion in the same tumor type increases matrix stiffening through collagen crosslinking, causing tumor cell dissemination and metastasis. These observations highlight the complex and paradoxical role of LOX family members in various types of cancer.(Saito et al, 2019)

LOX expression is found to be upregulated by HIF-1α and is required for hypoxia-induced metastasis in human breast and head and neck tumors.(Cano et al., 2012) Secreted LOX and hypoxia are relevant to metastasis and premetastatic niche formation in breast cancer (Canesin et al., 2015). It has been shown that the LOX expression is high in the OSCC tissues associated with areca nut chewing habit (Fillies et al., 2005). HIF-1α expression is high in OSCCs specimens from areca quid chewers (Kober et al., 2018 ). Thus, there is a need for better understanding the exact pathways by which areca nut extracts upregulate LOXL-2 in hypoxic tumor microenvironment. The present study was undertaken to study the association of HIF-1α with LOXL-2 in OSCC and their role in tumor progression.

## Materials and Methods

Study group- This study was performed on 90 histopathologically proven OSCC biopsied tissues after taking written informed consent from patients. The study was approved by institutional ethical committee of Maulana Azad Institute of Dental Sciences. Duration and quantity of areca nut chewing habit were recorded. TNM staging was done according to AJCC, 7^th^ edition. All biopsy tissues were histopathologically graded according to Anneroth’s system (1987).

Antibodies and immunohistochemistry- 2–4μ thick paraffin embedded sections were used for immunohistochemistry (IHC). Sections were deparaffinized and hydrated followed by antigen retrieval at 100°C for 20 minutes in EDTA-Tris buffer. Primary antibody incubation was done for one hour with HIF-1α Antibody (NB100-123, Novus Biologicals) at 1:100 dilution and for 16 hours with LOXL-2 Antibody (NBP1-32954, Novus Biologicals) at 1:100 dilution. Master Polymer Plus Detection System, HRP (Master diagnostics), and Poly HRP Secondary Detection System (Dako, Denmark) were used for HIF-1α and LOXL-2 staining. Clear cell variant of Renal Cell Carcinoma and liver fibrosis tissue sections were taken as a positive control for the expression of HIF-1α and LOXL-2, respectively.

**Figure 1:**
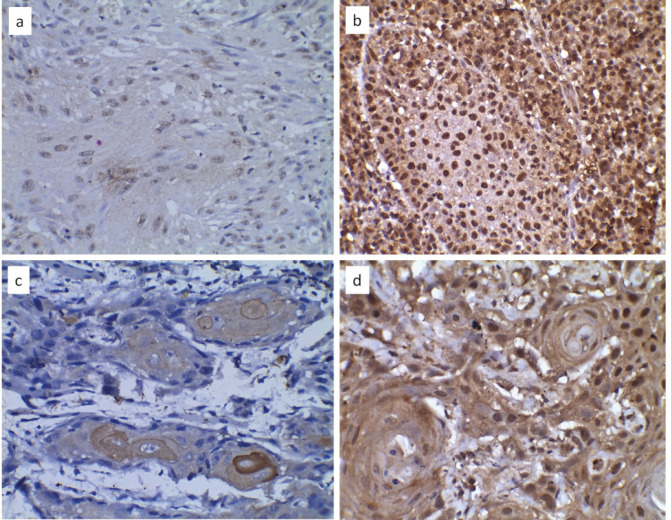
HIF-1α Immunoexpression: a- staining extent 3 and intensity of 1, b- staining extent 3 and intensity of 3. LOXL-2 immunoexpression: c- staining extent 2 and intensity of 2, d- staining extent 3 and intensity of 3. (40X)

**Figure 2:**
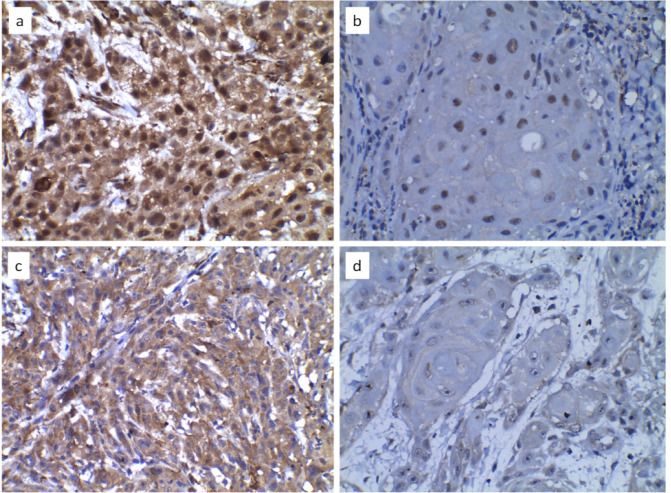
a- Nuclear and cytoplasmic staining are strong (A), b- Nuclear staining is strong, cytoplasmic staining is weak (B), c- Nuclear staining is weak, cytoplasmic staining is strong (C), and d-Both nuclear and cytoplasmic staining are weak (D). (40X)

Analysis of IHC reactions- Ten randomly selected high power fields were studied under a light microscope (Motic BA210, China) to assess the percentage of tumor cells stained and intensity of staining for both HIF-1α and LOXL-2. The percentage of immunopositive cells was scored as 0 (0% of cells stained), 1 (1%–25% of cells stained), 2 (26%–50% of cells stained), and 3 (51%–100% of cells stained). The staining intensity was scored as 0 (negative), 1 (weakly positive), 2 (moderately positive), and 3 (strongly positive).(Figure 1) An average of the product of the intensity score and the extent of staining score was used as the final staining score. Cut-off scores used for high HIF-1α and LOXL-2 staining were >6 and >4, respectively, as described in previous studies. The cellular localization of the LOXL-2 immunostaining was recorded as (A) Strong nuclear and cytoplasmic staining, (B) Strong nuclear, weak cytoplasmic staining, (C) Weak nuclear, strong cytoplasmic staining, and (D) Weak nuclear and cytoplasmic staining (Figure 2).

Statistical analysis- SPSS software version 21 was used for statistical calculations. Differences between clinico-pathologic variables and IHC results were analyzed using Kruskal–Wallis test, Chi-square test and Mann–Whitney test. Pearson’s correlation coefficient was carried out to determine correlation between the HIF-1α and the LOXL-2 immunoreactivity. P value ≤ 0.05 was considered to be statistically significant.

## Results

The study group consisted of 83.3% males and 15.55% females, with an age range of 12–77 years (mean age = 49.5 years). Site of the lesion was alveobuccal complex in 38.9%, alveolar mucosa in 25.5%, bucaal mucosa in 17.8%, tongue in 12.2%, and palate in 5.5% cases. Maximum number of lesions presented as ulcer (32.2%) or ulceroproliferative growth (32.2%), followed by endophytic lesions (30%), and exophytic or white patch (4.5%). 70% cases were areca nut chewers. 43.3% cases showed high HIF-1α expression. 72.2% cases showed high LOXL-2 expression. 

The HIF-1α expression and the LOXL-2 expression were compared with the tumor category, nodal stage, clinical TNM stage, and histological grade using Kruskal–Wallis test (Table I). There was significant difference between HIF-1α immunoexpression and tumor stage (p-value = 0.008), but no significant difference between LOXL-2 expression and tumor stage. Mann–Whitney test showed significant difference in HIF-1α expression between T1 and T2 (p- value= 0.04), T1 and T3 (p- value=0.011) and T1 and T4a (p- value= 0.03). High HIF-1α expression was noted in T1-staged tumors. No significant difference was noted between T2 and T3, T2 and T4a, and T3 and T4a. Also, no significant differences were found for HIF-1α expression and LOXL-2 expression when compared with nodal stage, clinical TNM stage, and histological grade. There was no significant difference (p-value=0.162) between LOXL-2 expression and areca nut chewing habit.

**Figure 3:**
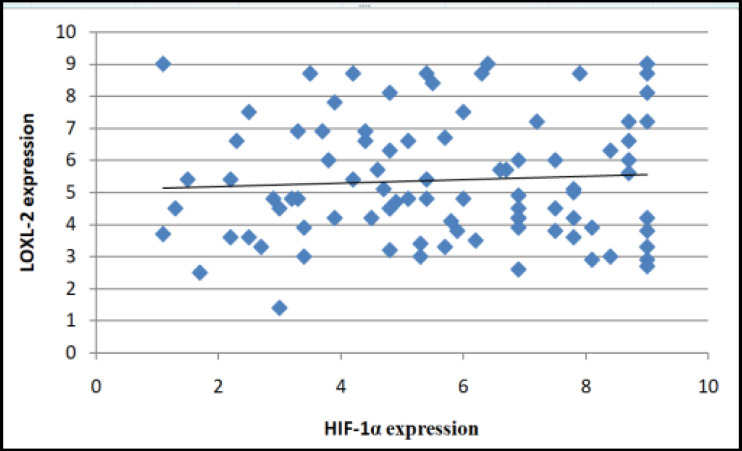
Correlation between *HIF-1α* and *LOXL-2* Expression in OSCC Cases

Localization of LOXL-2 staining was compared with the tumor category, nodal stage, clinical TNM stage, histological grade, and areca nut chewing habit using Chi-square test (Table II). There were significantly higher number of cases with strong cytoplasmic staining (C) in N0 and N1 stage when compared with N2 stage. No significant differences were found when localization of LOXL-2 staining was compared with tumor stage, clinical TNM stage, histological grade, and areca nut chewing habit. A positive Pearson’s correlation between HIF-1α and LOXL-2 immunoexpressions was present to some extent, though a statistically significant value was not met (p= 0.06) (Figure 3).

**Table 1:** Comparison of Clinicopathological Parameters with the HIF-1α and LOXL-2 Immunoexpressions Using Kruskal-Wallis Test

Clinico-Pathological Parameter		No. of cases	HIF-1α expression		LOXL-2 expression	
			Mean rank	p- value	Mean rank	p-value
Tumor stage (T)	Total	90		0.008*		0.968
	T1	21	62.6		45.5	
	T2	33	41.29		46.68	
	T3	8	38.62		43.31	
	T4a	28	39.61		44.73	
Nodal stage (N)	Total	90		0.821		0.988
	N0	31	43.18		46.19	
	N1	42	45.13		43.99	
	N2a	4	57.62		47.12	
	N2b	10	50.45		48.2	
	N2c	3	42		48.33	
Clinical Stage	Total	90		0.095		0.979
	I	12	57.25		46.42	
	II	13	32.15		48.04	
	III	28	48.68		45.23	
	IVa	37	43.97		44.51	
Histological grade	Total	90		0.072		0.263
	I	38	50.17		47.39	
	II	38	38.18		40.76	
	III	14	52.68		53.21	

*, indicates significant result

**Table 2:** Comparison of Cellular Localization of LOXL-2 Immunoexpression with Clinicopathological Parameters

Clinico- Pathological Parameters		No. of cases	A -17	B -2	C -33	D -38	P-value
Tumor stage (T)	Total	90					0.682
	T1	21	7	0	5	9	
	T2	33	4	1	14	14	
	T3	8	2	0	2	4	
	T4a	28	4	1	12	11	
Nodal stage (N0)	Total	90					0.038*
	N0	31	6	2	16	7	
	N1	42	4	0	15	23	
	N2a	4	2	0	0	2	
	N2b	10	3	0	2	5	
	N2c	3	2	0	0	1	
Clinical Stage (TNM)	Total	90					0.511
	I	12	3	0	5	4	
	II	13	3	1	7	2	
	III	28	4	0	9	15	
	IVa	37	7	1	12	17	
Histological Grade	Total	90					0.518
	I	38	8	1	14	15	
	II	38	4	1	14	19	
	III	14	5	0	5	4	
Areca nut Habit	Total	90					0.097
	Present	63	15	2	24	22	
	Absent	27	2	0	9	16	

*, indicates significant result

## Discussion

Squamous Cell Carcinoma (SCC) is an aggressive solid tumor which grows rapidly, because of which the simultaneous neoangiogenesis occurs to meet the oxygen demands and central part of the tumor might remain hypoxic due to less orderly vasculature and necrosis. HIF-1α is the master regulator of many angiogenic factors like VEGF and VEGFR (Semenza, 2003). Molecular processes facilitating tumor survival and spread such as deﬁciency or mutation of tumor suppressor genes (*Von Hippel-Lindau, p53*, and* phosphatase and tensin homolog*), and ampliﬁcation of oncogenes (Akt, Ras) were shown to be associated with HIF-1α overexpression in previous studies (Munipalle et al., 2011). Recently identified another regulator of hypoxia-induced tumor growth is LOXL-2. LOXL-2 has two domains: a cytokine receptor-like domain and four scavenger-receptor cysteine-rich domains. Other proteins having these domains seem to be involved in cell adhesion and EMT.(Lucero and Kagan, 2006)

Eckert AW et al found that 42.7% cases were having negative or weak HIF-1α expression whereas 57.3% cases had moderate to strong HIF-1α expression in OSCC tissues.(Eckert et al., 2010) Our results are in contrast to the above study, which can be attributed to the non-static nature of hypoxic areas in OSCC (Eckert et al., 2012). High LOXL-2 expression in larger proportion of cases (72%) in the present study can be explained by the fact that there are alterations in the gene expression patterns of collagen crosslink associated enzymes in oral, head, and neck cancers. Further, LH-2 mediated stable covalent cross-links of collagen, which are catalyzed by LOXL-2, are increased in OSCC (Sakai et al., 2009).

T1-staged tumors exhibited high *HIF-1α *expression in the present study. Expression of *HIF-1α* is an early event in tumorigenesis (Eckert et al., 2012). Ovarian cancers show HIF-1α in early stages, if it is not associated with any other genetic mutation (Semenza, 2003). As described by Kappler et al., (2018) HIF-1α is always increased in an environment with acidic pH that could also result by other metabolic disturbances in the tumor microenvironment, other than hypoxia. Perfusion-restricted hypoxia (also called acute hypoxia) mainly mediated by HIF-1α causes constant fluctuations leading to cyclic periods of hypoxia and re-oxygenation that can lead to the development of a heterogeneous cell population within the tumor. Diffusion-restricted hypoxia (also called chronic hypoxia) mainly mediated by HIF-2α refers to the sustained restriction in oxygen diffusion by abnormal vascular network (Nejad et al., 2021) LOXL2 coexpress Snail1 and is related to Twist transcription in breast cancer, thereby may be intimately involved in the process of EMT. Our study showed no statistically significant difference in LOXL-2 expression in different tumor stages in accordance with some previous studies in esophageal SCC (Tilakaratne and Nissanka, 2011; Li et al, 2012).

N0 stage showed higher number of cases with low HIF-1α expression, but the differences were not statistically significant. Fillies T et al and Lin PY et al also noted no significant association of HIF-1α expression with nodal stage in SCC of floor of the mouth and oral cavity, respectively (Fillies et al, 2005; Lin et al, 2008). In hypoxic microenvironment tumor cells stimulate the generation miR-21-rich exosomes that are passed on to normoxic cells and enhance prometastatic behaviors. Also, exosomes generated from hypoxic oral squamous cell carcinoma (OSCC) cells stimulate the migration and invasion of tumor cells in a HIF-1α and HIF-2α-dependent manner (Norouzian and Balouchi-Anaraki, 2019).

In all the nodal stages, more number of cases showed high LOXL-2 expression in our study, though the results were non-significant. LOXL-2 catalyzed crosslinks have been shown to be positively correlating with advanced stage tumors and lymph node metastasis in OSCC (Saito et al., 2019).

Fillies T et al found no significant association of HIF-1α expression with TNM stage in SCC of floor of the mouth, which is in accordance with our study. They speculated that expression of HIF-1α is not hypoxia related, and is related to alterations in oncogenes and tumor suppressor genes (Fillies et al., 2005). When *HIF-1α *overexpression was concomitantly present with p53 mutation in ovarian carcinoma, statistically significant decrease in overall survival was observed. Therefore, the effect of *HIF-1α* overexpression might be dependent on the cancer type and presence or absence of other genetic alterations (Semenza, 2003). No significant correlation was noted between *LOXL-2* expression and TNM factors. It was hypothesized that possibly, both genetic and epigenetic mechanisms are involved in the modification of *LOXL2* gene expression during cancer progression (Zhan et al., 2012).

The statistical difference of *HIF-1α* and *LOXL-2 *expressions within different histological grades was not significant. Similarly, Fillies T et al also observed no significant association of HIF-1α expression with histological grade in SCC of floor of the mouth (Fillies et al., 2005). The results of the study conducted by Ahn S G et al in breast cancer patients were similar to our study showing no significant difference between LOXL-2 immunoexpression and different histological grades (Ahn et al., 2013).

Li et al., (2012) observed that normal esophageal squamous epithelial cells showed strong nuclear staining for LOXL-2 and no cytoplasmic staining. Restrained nuclear staining and enhanced cytoplasmic staining were noted in ESCC cells which significantly correlated with, larger tumors, increased lymph node metastasis, and tumor stage III. Overall survival of the patients with high nuclear expression of LOXL-2 was significantly more when compared to patients with low/negative nuclear expression. These results indicated that nuclear LOXL-2 might have some protective role against esophageal SCC.Altered tissue architecture in tumors affecting mammary and gastric glands leads to interaction between membrane bound LOXL-2 and tumor matrix, facilitating metastatic behavior (Hollosi et al., 2009). Any alteration in cellular localization of LOXL-2 expression, whether it is from membranous to cytoplasmic or nuclear to cytoplasmic might be a prognostic sign depending on the organ affected by cancer. The present study is probably the first of its kind to analyze the possible alterations in cellular localizations of LOXL-2 expression in OSCC in great detail.

In present study, maximum number of cases were labeled as ‘D’ (42.2%) followed by ‘C’ (36.6%), ‘A’ (18.9%), and ‘B’ (2.2%).(Table II). It was found that when cellular localization was compared with nodal stage, the difference was statistically significant with a p- value of 0.038 whereas, all other parameters like tumor stage, clinical stage, histological grade, and areca nut chewing habit did not show any statistically significant difference. Few cases also exhibited membranous staining at focal areas, most of them with well-differentiated morphology. Peinado et al have speculated that LOXL-2 residing in the cytoplasm inhibits GSK3-induced Snail degradation, eventually leading to upregulation of Snail and stimulation of epithelial mesenchymal transition (Li et al, 2012; Hollosi et al, 2009). These results indicate that presence of *LOXL-2* in the cytoplasm might have a role in carcinogenesis and thus cytoplasmic expression was noted to be more frequent in OSCC cases in the present study . 

Shieh et al., (2007) found an upregulation of LOX and *LOXL-2* mRNA expressions in areca associated OSCC tissues and cell lines relative to their normal counterparts. As lysyl oxidase is a secreted copper-dependent enzyme and areca nut also contains a high amount of copper in soluble form, the increased level of soluble copper found in oral fluids of chronic areca nut chewers may relate to pathogenesis of SCC through LOX activation.In accordance with the above quoted study, greater proportion of patients expressed high LOXL-2 expression who had history of consuming areca nut when compared to those who did not have areca nut chewing habit. But, the difference was not statistically significant.

Correlation analysis was performed to assess the relationship between HIF-1α and LOXL-2 immunoexpressions in OSCC. The results showed a positive correlation, which was not statistically significant. Erler JT et al showed that HIF-1α-induced LOX mediates the process of EMT suggesting a synergic role of both *HIF-1α *and *LOXL-2* in cancer progression, but the exact crosstalk is not known. They suggested that secreted LOX is responsible for the invasive properties of hypoxic human cancer cells through focal adhesion kinase activity and cell to matrix adhesion ( Erler et al., 2006).

To conclude, *HIF-α* overexpression was seen in T1-staged tumors indicating its role in early tumorigenesis. Cytoplasmic *LOXL-2* expression was significantly different in different nodal stages, which may play a role in the progression of OSCC.
